# Achilles Enthesitis in Psoriatic Arthritis: Inter-Observer Reliability of Ultrasound Findings

**DOI:** 10.3390/jcm14248738

**Published:** 2025-12-10

**Authors:** Mihaela Agache, Luminita Enache, Claudiu Costinel Popescu, Bianca Dumitrescu, Catalina Elena Ionescu, Denisa Elena Moscalu, Anca Bobirca, Catalin Codreanu

**Affiliations:** 1 Rheumatology Department, “Carol Davila” University of Medicine and Pharmacy, 020021 Bucharest, Romania; mihaela.agache@reumatologiedrstoia.ro (M.A.); claudiu.popescu@reumatologiedrstoia.ro (C.C.P.); bianca.dumitrescu@reumatologiedrstoia.ro (B.D.); catalina.ionescu@reumatologiedrstoia.ro (C.E.I.); denisa.moscalu@reumatologiedrstoia.ro (D.E.M.); anca.bobirca@umfcd.ro (A.B.);; 2 “Ion Stoia” Clinical Center for Rheumatic Diseases, 020983 Bucharest, Romania; 3 Rheumatology Department, “Ioan Cantacuzino” Clinical Hospital, 011437 Bucharest, Romania

**Keywords:** psoriatic arthritis, ultrasound, enthesitis

## Abstract

**Background/Objectives**: Enthesitis is a hallmark feature across the spondylarthritis spectrum, including psoriatic arthritis (PsA). In recent years, advanced imaging techniques, particularly musculoskeletal ultrasound (MSUS), have demonstrated higher sensitivity than clinical examination in detecting enthesitis. This study aimed to evaluate the inter-observer agreement for the diagnosis of Achilles enthesitis in a cohort of PsA patients. A secondary objective was to explore specific ultrasound diagnostic criteria for identifying active, inflammatory enthesitis in this population. **Methods**: Adult patients with PsA, all fulfilling CASPAR classification criteria, were recruited and underwent both clinical and ultrasonographic assessment of the bilateral Achilles tendons. Each patient was scanned by 4 rheumatologists in a direct study, followed by a blinded evaluation of static images of the same patients. The examiners assessed the presence of enthesitis components according to the OMERACT criteria. In addition, the images were subsequently evaluated by 10 MSUS-experienced rheumatologists who were asked to classify the enthesitis as inflammatory by selecting one of the following responses: “yes”, “no,” or “possible”. **Results**: Ten PsA patients, with a median age of 60 and a median DAPSA score of 21, were included. Both direct and image-based inter-observer studies showed high agreement values for enthesophytes (κ > 0.6), erosions (κ > 0.5), and entheseal thickness (κ > 0.5). In both, low agreement was observed for hypoechogenicity (κ between 0.1 and 0.4). Erosions and power Doppler (PD) signal in erosions showed statistically significant differences between the “possible” and definite (“yes”) inflammatory enthesitis groups. A PD signal of grade 2 or 3 within the enthesis or erosions was observed exclusively in cases classified as definite (“yes”) inflammatory enthesitis. Similarly, a grade 3 PD signal in the bursa was found only in patients with definite inflammatory enthesitis. This study proposes a novel ultrasound scoring system for defining inflammatory enthesitis. The score demonstrated overall good diagnostic performance, with a sensitivity of 67% and a specificity of 100%. **Conclusions:** The relatively low inter-observer agreement regarding hypoechogenicity and the presence of PD highlights the need for targeted educational interventions to improve interpretation in MSUS. Erosions and PD signal within erosions appear to be significant discriminatory features for identifying inflammatory enthesitis.

## 1. Introduction

Enthesitis is a hallmark feature across the spondylarthritis spectrum, including psoriatic arthritis (PsA). In recent years, due to advances in disease pathogenesis and the emergence of new treatments, there has been increasing research interest in this domain [1,2,3]. The identification of enthesitis supports diagnosis and monitoring of disease activity and guides treatment decisions in PsA [4].

New imaging techniques, particularly musculoskeletal ultrasound (MSUS), which has been shown to have higher sensitivity compared to clinical examination, aim to clarify the role of enthesitis in the diagnosis and management of PsA [5].

In 2014, a preliminary definition of enthesopathy was proposed by OMERACT (Outcome Measures in Rheumatology) [6]. This was updated in 2018 through a consensus among MSUS experts [7]. The OMERACT consensus group defined the elements of enthesitis as follows: structural features (erosions, enthesophytes, calcifications) and inflammatory features (hypoechogenicity, thickening, presence of power Doppler (PD) signal). Standardization in the acquisition and interpretation of ultrasound images has led to improved agreement between different examiners over the past decade [8].

EULAR (European Alliance of Associations for Rheumatology) recommends the use of ultrasound to detect peripheral enthesitis in support of the spondylarthritis diagnosis [9]. These recommendations also include patients with PsA, although the guidelines do not clearly distinguish between the two entities. There are also recent proposals for early PsA [10], highlighting that imaging abnormalities should be interpreted with caution, especially when they do not match clinical findings. The diagnosis of PsA should be based not only on ultrasound but on a comprehensive evaluation that includes a combination of clinical, laboratory, and imaging findings.

Although MSUS is an accessible, fast, and non-irradiating tool, its utility is constrained by operator dependency and variability in equipment technical performance, particularly when assessing vascularization through PD [8]. The evaluation of enthesitis in clinical studies requires consistent agreement on the defining features; however, in PsA, there is limited published data in the scientific literature on this subject. In 2022, a large multicenter study assessed ultrasound inter-observer reliability of enthesitis referring to spondylarthritis in general [11] and demonstrated good correlation with the OMERACT definitions for bone erosions, enthesophytes, calcifications, and PD signal at entheses. However, inter-observer agreement was found to be low for entheseal thickness and hypoechogenicity, suggesting the need to revise the definitions of these two major components of the current ultrasound definition [11]. According to OMERACT [12] and EFSUMB [13] recommendations, the PD signal is an essential feature for assessing active enthesitis, particularly in PsA or spondylarthritis [7]. Firstly, the accuracy of PD depends on the quality of the equipment (high-performance machines are required), as well as the examiner’s expertise and experience [14]. The PD technique has high specificity but low sensitivity, which makes it highly useful for confirming a diagnosis but inadequate for ruling it out when the signal is absent. In a comprehensive meta-analysis, Bibas et al. [15] reported a PD specificity of 97.9% and sensitivity of 14.7% for PsA-related enthesitis. Hallstrom et al. [16] remarked that even with experienced operators, subjective factors may influence PD assessments.

The Achilles tendon is a reference anatomical site for ultrasound evaluation of entheses, being included in all enthesitis scoring systems [17,18]. It is the most frequently affected site in PsA, where PD activity and erosions are the most discriminative features compared to healthy individuals (rarely seen in non-PsA patients) [15].

The increasing accessibility of imaging assessment of entheses, along with the development of scoring systems, has also led to the identification of entheseal changes in non-inflammatory conditions. Recent studies have reported features of active enthesitis in non-inflammatory contexts, for example, in marathon runners [19] and patients with high body mass, likely due to mechanical stress [20]. Additionally, other demographic factors, such as age, are associated with the severity of ultrasound abnormalities in joints and entheses in healthy individuals without musculoskeletal symptoms [19].

In PsA, the detection of active enthesitis is important for diagnosis, especially for early detection of the disease (in subclinical stages), but also for prognosis and treatment monitoring [10]. Ultrasound-detected entheseal abnormalities, such as bone erosions and PD signal, have been associated with structural joint damage and may serve as biomarkers of disease severity [21]. Recent research has focused on identifying and combining specific features to increase the diagnostic specificity for inflammatory enthesitis (for example, integrating PD grading with the presence of structural abnormalities). In addition to PD, the presence of an erosion is now increasingly recognized as a second key ultrasound marker [22]. Regarding treatment, enthesitis shows response to biologics but slower compared to synovitis [22].

## 2. Objectives

This study aimed to evaluate, within a group of rheumatologists who routinely perform MSUS, the inter-observer agreement for the diagnosis of Achilles enthesitis according to the OMERACT definition at the Achilles tendon insertion site in a group of PsA patients. A second objective was to explore specific ultrasound diagnostic criteria for detecting active, inflammatory enthesitis in PsA patients.

## 3. Materials and Methods

### 3.1. Patients

**Figure 1:**
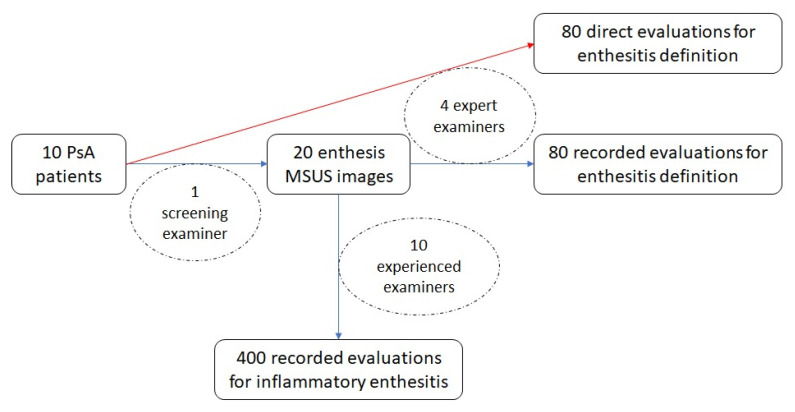
Study design.

Adult patients diagnosed with PsA, all fulfilling the CASPAR (ClASsification criteria for Psoriatic ARthritis) [23], who attended the outpatient clinic, were interviewed and underwent both clinical and ultrasonographical assessment of the bilateral Achilles tendon on five randomly selected days in November 2024 by the same screening examiner (C.E.I.) who was not included in the inter-observer study. Exclusion criteria were recent trauma in the lower limb, surgical interventions on the lower limb, or injections at the enthesis or adjacent structures within the preceding six months, as well as initiation or modification of conventional synthetic or biologic disease-modifying antirheumatic drugs (cs/bDMARDs) and/or glucocorticoid therapy during the same period. To ensure randomization, on each study day, the first two eligible patients presenting with symptoms related to the Achilles tendon and/or clinical signs of inflammation were subsequently scanned on the same day by four other examiners (M.A., L.E., B.D., and D.E.M.), all with expert-level experience in MSUS and trained in the OMERACT enthesitis definition. The participating examiners were blinded to the findings or interpretations of the other assessors. This evaluation is referred to as the “direct inter-observer study” for enthesitis definition, where patients were examined directly at the time of presentation. In December 2024, the same four examiners also assessed the original ultrasound images obtained by the screening examiner; this part is referred to as the “recorded inter-observer study” for enthesitis definition. The original ultrasound images obtained by the screening examiner were also reviewed for the diagnosis of inflammatory enthesitis by ten other rheumatologists with at least five years of experience and training in MSUS; this part is referred to as the “recorded inter-observer study” for inflammatory enthesitis (Figure 1).

The study was approved by the local Ethics Committee (No. 10172/08.11.2024) and conducted in accordance with the principles of the Declaration of Helsinki. All patients provided written informed consent.

### 3.2. MSUS

All ultrasound assessments in the direct inter-observer study were performed in accordance with EULAR recommendations [24], utilizing a GE LOGIQ E10 machine (GE Healthcare, Chicago, IL, USA; grayscale (GS) and PD, 4–20 MHz linear probe). Thus, each patient underwent 4 examinations of the same enthesis. Also, there were recorded images of each enthesis. Each image was centered on the enthesis, with measurement of entheseal thickness at the insertion, perpendicular to the tendon surface, and the distance from the PD signal to the cortical bone was recorded according to EULAR guidelines for image acquisition in rheumatology [24].

For the ultrasound assessment of the Achilles tendon insertion, the patient was placed in the prone position, with the foot relaxed at the edge of the examination table. Scanning was performed using high-frequency transducers in both GS and PD modes, in both longitudinal and transverse planes, both static and dynamic.

In both the direct and image-based inter-observer studies for enthesitis definition, the examiners—blinded to patients’ clinical data—were instructed to assess the presence or absence of each item of the OMERACT ultrasound definition of enthesitis: hypoechogenicity, thickened and PD signal. Additionally, the presence of PD signal in the Achilles tendon (outside the enthesis) and the presence of retrocalcaneal bursitis, with or without PD signal, were also assessed. Each of the above features was scored as “present” or “absent”. In the recorded inter-observer study for inflammatory enthesitis, each image was assessed for the diagnostic features of enthesitis, entheseal thickening, hypoechogenicity, enthesophyte, calcification, bone erosions, and bursitis. PD signals in enthesis, erosion and bursa were graded on a semiquantitative scale (grade 1 = minimal, grade 2 = moderate, grade 3 = severe). Each evaluating physician was required to interpret and classify each of the entheseal images and decide whether they represented inflammatory enthesitis by selecting one of the following responses: “yes”, “no,” and “possible.”

### 3.3. Statistics

The normality of data distribution was assessed using descriptive statistics, normality plots, and the Kolmogorov–Smirnov test with Lilliefors correction. Continuous variables were reported as “median (minimum–maximum)” due to non-normal distribution, while dichotomous variables were reported as “observed frequency (percentage of subgroup).” The frequency of ultrasound findings represents the average frequency of positive results across evaluators. Inter-observer reliability was estimated by calculating the kappa coefficient using Light’s method, with 95% confidence intervals (CI, representing the 2.5 and 97.5 percentiles from 1000 random iterations of the kappa values). For the inflammatory enthesitis score, Cohen’s kappa was used. Kappa coefficients were interpreted according to Landis and Koch [25]. Associations between categorical variables were analyzed using χ^2^ independence tests, with examination of adjusted standardized residuals (ASR) and post hoc analysis with Bonferroni correction of statistical significance thresholds. The statistical tests were considered significant if *p* < 0.05 and were performed using IBM SPSS Statistics version 25.0 for Windows (IBM Corp., Armonk, NY, USA). Post hoc reliability analyses for the kappa statistics (4 raters for direct assessments of binary OMERACT items and 10 raters for recorded readings of the three-category inflammatory enthesitis), using a hypothesis-testing framework (alpha 0.05, power 0.80) with κ = 0.40 for the null hypothesis versus κ = 0.60 for the alternative hypothesis, and conservative marginal distributions (binary prevalence 0.50; three-category proportions 0.50/0.30/0.20), estimated a requirement of approximately 25 entheses for the direct study and 14 entheses for the recorded study (subjects rated by all raters). In a precision framework, the expected 95% CI half-width around κ = 0.60 was approximately 0.11–0.14 (direct study) and 0.07–0.10 (recorded study). Calculations followed Donner & Rotondi’s methodology [26] and were generated with R’s kappaSize package (v. 4.5.2 for Windows).

## 4. Results

### 4.1. Inter-Agreement Study for Enthesitis Definition

This study included 10 PsA patients, 6 men and 4 women, with a median age of 60 years (range 51–71) and a median Disease Activity Index for Psoriatic Arthritis (DAPSA) score of 21 (range 4–54). Regarding treatment, 80% of patients were on csDMARDs, primarily methotrexate (70%), while 70% were treated with bDMARDs, including adalimumab, etanercept, and ixekizumab (2 patients each) and secukinumab (one patient). None of the patients was receiving glucocorticoids at the time of the assessment. All patients had been on stable treatment for at least 6 months prior to evaluation.

In the direct inter-observer study for enthesitis definition, performed using the GE ultrasound machine (Table 1), Achilles tendon enthesitis was identified in 58.8% of evaluations (47 out of 80 entheses examined), and PD activity was reported in 23.8% of cases. Overall, inter-observer agreement among physicians was moderate for the diagnosis of enthesitis and the detection of PD signal. Agreement was very good (κ > 0.6) for the following features: thickening, erosions, enthesophytes, and normal enthesis appearance. In contrast, agreement was low for the detection of hypoechogenicity.

In the recorded inter-observer study for enthesitis definition, Achilles tendon enthesitis was identified in 51.3% of evaluations (41 out of 80 entheses examined), with moderate agreement, while PD activity was observed in 25.0% of assessments (20 out of 80 entheses examined), with substantial agreement among evaluators. Very good agreement (κ > 0.6) was achieved for the identification of the following features: erosions and enthesophytes (Table 2). Agreement was low for the detection of hypoechogenicity and calcifications.

**Figure 2:**
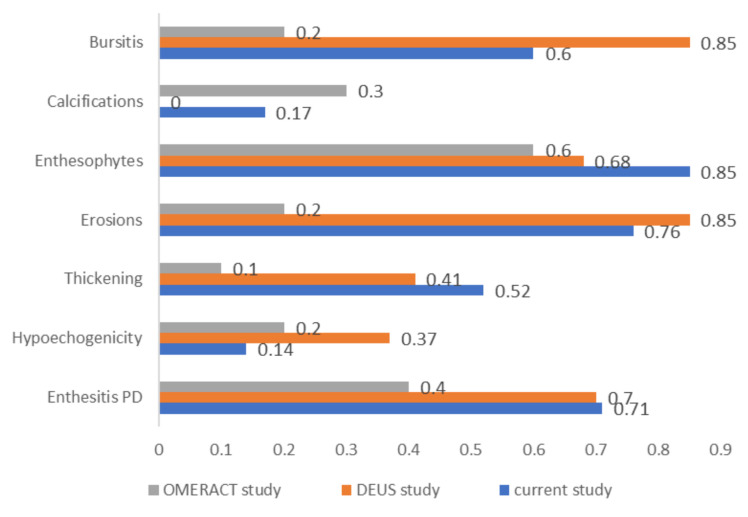
Agreement data (interpretation of κ coefficients) for the component elements of enthesitis: comparison of the present study with two other studies mentioned in the “Discussion” chapter [27,28].

A comparison of agreement coefficients observed in this study with those reported by two major similar studies is depicted in Figure 2.

### 4.2. Inter-Observer Study for Inflammatory Enthesitis

**Figure 3:**
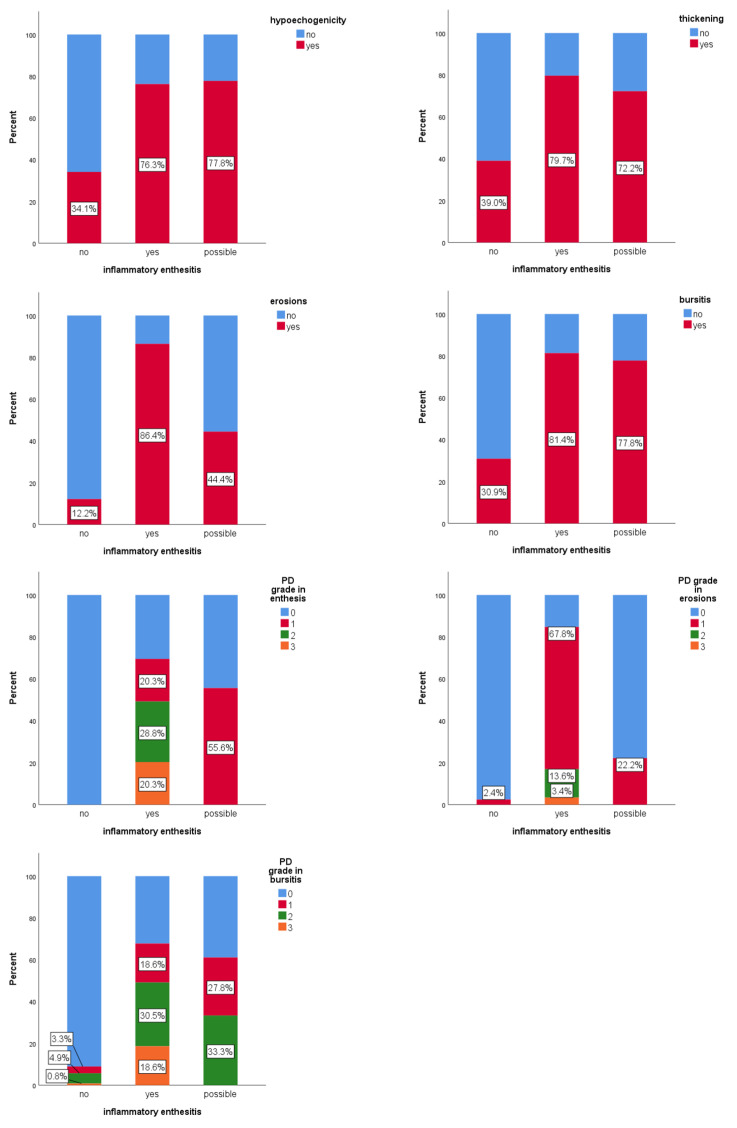
The prevalence of hypoechogenicity, thickening, erosions, bursitis, and PD signal grades (in enthesis, erosions and bursitis) among cases labeled with possible or definite inflammatory enthesitis.

Of the 20 entheses evaluated for inflammatory enthesitis (Figure 3), full consensus (100% agreement) among all ten examiners was achieved in only two cases, both of them classified as inflammatory active enthesitis. For eight entheses, there was full agreement that the image did not represent active inflammatory enthesitis. Across all evaluations (200 answers), 61.5% of images were classified as without enthesitis, 29.5% as confirmed active inflammatory enthesitis, and 9% of images as “possible” inflammatory enthesitis. PD signal was detected in 9.8% of the evaluations in which enthesitis was not diagnosed. It was observed that 84.4% of cases, classified as definite inflammatory enthesitis, displayed at least four features from the OMERACT definition of enthesitis.

Hypoechogenicity was significantly associated with active inflammatory enthesitis (χ^2^(2) = 34.2, *p* < 0.001). The “possible” category of enthesitis had a similar frequency of hypoechogenicity to the “confirmed” category (77.8% vs. 76.3%; *p* = 0.9), although the statistical strength of the association was weaker (ASR ± 2.4 vs. ± 5.8). Thickening at tendon insertion was also significantly associated with active inflammatory enthesitis (χ^2^(2) = 29.2, *p* < 0.001). The “possible” and confirmed categories (“yes”) had similar frequencies (72.2% vs. 79.7%; *p* = 0.5). Erosions were significantly associated (χ^2^(2) = 94.8; *p* < 0.001) with inflammatory enthesitis. The “possible” category showed significantly fewer erosions than the confirmed (“yes”) group (44.4% vs. 86.4%; *p* < 0.001), but with a weaker effect. Bursitis was also significantly associated (χ^2^(2) = 46.7, *p* < 0.001) with active inflammatory enthesitis. Frequency was similar between “possible” and “confirmed” categories (77.8% vs. 81.4%; *p* = 0.7). PD signal in the enthesis showed a significant association (χ^2^(3) = 148.2, *p* < 0.001) with active enthesitis. Although grade 1 was more frequent in the “possible” group (55.6%) than in the confirmed group (“yes”) (20.3%), this difference was not statistically significant (*p* = 0.3). Grades 2 and 3 were found only in confirmed (“yes”) enthesitis. PD signal in erosions was also significantly associated (χ^2^(3) = 134.3; *p* < 0.001) with active enthesitis. Grade 1 PD signal in the “possible” group was significantly lower than in the confirmed group (“yes”) (22.2% vs. 67.8%; *p* < 0.001). PD signal in the bursa was significantly associated (χ^2^(3) = 82.5; *p* < 0.001) with inflammatory enthesitis, though grades 0–2 did not differ significantly between “possible” and “confirmed” groups (*p* > 0.2).

**Figure 4:**
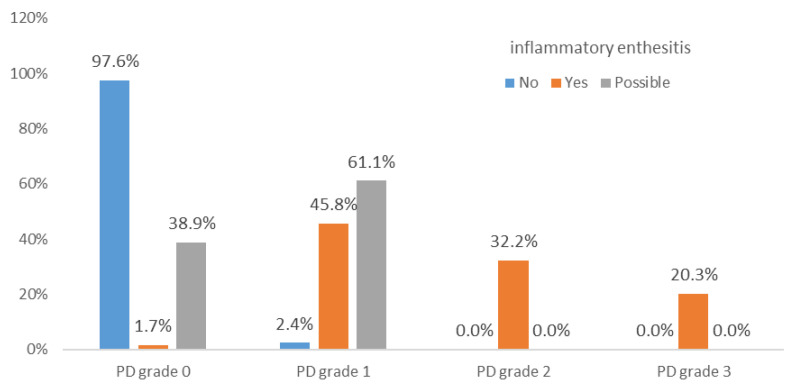
Percent distribution across the three inflammatory enthesitis categories (“yes,” “no,” “possible”) for combined PD signal (in enthesis or erosion—highest score among the two).

PD grades 2 and 3, in the enthesis, erosion, or combined (enthesis and erosion), occurred only in confirmed enthesitis cases. Grade 3 PD in the bursa was also present only in confirmed cases (Figure 4). PD in erosions may serve as a statistically significant differentiator for confirmed inflammatory enthesitis.

### 4.3. Inflammatory Enthesitis Score

Based on the above data, for a patient with an ultrasound (GS) diagnosis of enthesitis (i.e., presenting with either thickening or hypoechogenicity), we propose applying a score calculated using the following methodology.

The proposed ultrasound score is based on grading the OMERACT-defined elements as follows (Table 3):

In our cohort, grade 1 or 2 PD signal in the bursa was present in both definite and possible inflammatory enthesitis cases, indicating that such lower-grade findings may represent non-specific or mechanical changes. In contrast, grade 3 PD signal was observed exclusively in definite inflammatory enthesitis, suggesting a higher specificity for active disease. Therefore, for the purposes of the scoring system, we applied a cut-off of >2 for a positive PD signal in the bursa.

**Figure 5:**
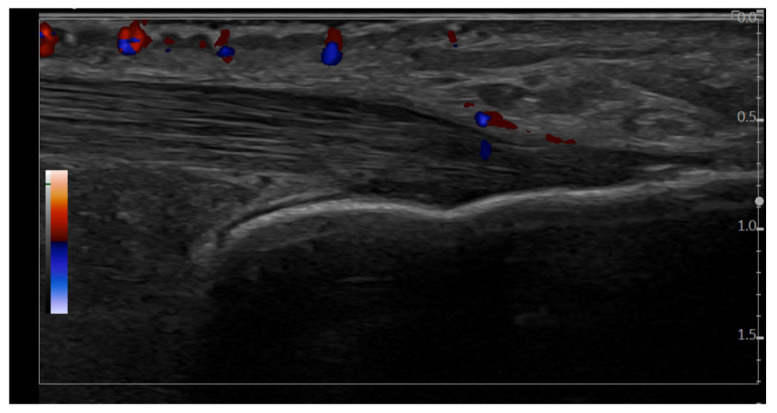
Longitudinal scan, in GS and PD mode, of the Achilles tendon insertion (GE LOGIQ E10 machine, 4–20 MHz linear probe). This conflicting image demonstrates a hypoechoic area at the entheseal level, which meets the GS OMERACT criteria for enthesitis. PD signal appears to be located within the tendon structure (4 evaluators considered it possible inflammatory enthesitis, while 6 evaluators considered it as not being inflammatory enthesitis).

To assess the validity of the proposed scoring system, its dichotomized evaluations (“yes” for score ≥ 2 and “no” for score 0 or 1) were compared against a reference standard derived from evaluations by 10 expert observers. For each of the 200 evaluated images, the reference standard was defined by majority vote: at least 6 out of 10 observers agreeing that the image shows inflammatory enthesitis, and at least 6 out of 10 observers obtaining a score of ≥ 2 on the proposed scoring system. In the case of ties (5 votes “yes”, 5 “no”), a conservative approach was taken—classifying the image as “no” to minimize false positives. This score has 67% sensitivity, 100% specificity, 85% accuracy, and Cohen’s Kappa = 0.687, reflecting substantial agreement, with statistically significant alignment (T = 3.237; *p* = 0.001). Cases of disagreement (for example, Figure 5) could reach consensus based on this standardized score.

## 5. Discussion

### 5.1. Standardization of Enthesitis Assessment

Two decades after the first ultrasound description of enthesitis, the OMERACT group defined by consensus in 2014 the basic ultrasound components of enthesitis, aiming for standardization in clinical studies [6]. OMERACT later validated this definition in 2018 [7], defining enthesitis as the presence of hypoechogenicity or increased thickness within a maximum of 2 mm from the bone, which may or may not be associated with a PD signal or other elements such as enthesophytes, erosions, or calcifications.

### 5.2. Inter-Observer Agreement

In the present study, substantial ultrasound inter-observer agreement was observed for structural changes, especially enthesophytes and erosions, and moderate for detecting PD signal and assessing entheseal thickness. Agreement was poor for hypoechogenicity and calcifications. This can likely be explained by the fact that hypoechogenicity is a subjective characteristic that requires dynamic tendon movement and is influenced by anisotropy at the bone insertion. Calcifications are often indistinguishable from enthesophytes, which is the reason why, in some studies, they are quantified together.

### 5.3. Comparison with Other Studies

The OMERACT initiative for defining enthesitis components [7] also published a validation study. This showed substantial agreement for enthesophytes (κ = 0.6) and moderate agreement for calcifications (κ = 0.3) and PD signal (κ = 0.4), while other features, such as erosions (κ =0.2), thickening (κ = 0.1), and hypoechogenicity (κ = 0.2), demonstrated lower levels of agreement. Overall, the κ values for most parameters in our study were higher than those reported in the OMERACT validation study.

Di Matteo et al. [11] conducted an international study on spondylarthritis using ultrasound images and clips (101 static images and 39 clips). The image-based study showed the following results: very good agreement for erosions and PD signal in the enthesis (κ = 0.85 and 0.70, respectively), followed by substantial agreement on structural changes like enthesophytes and calcifications (κ = 0.68). Agreement was moderate for entheseal thickening (κ = 0.41) and low for hypoechogenicity (κ = 0.37). The level of agreement was not influenced by the type of image format (clip or static image). The present study recorded similar results for PD signal detection, comparable values for erosions and enthesophytes, and limited reproducibility of hypoechogenicity (Figure 1).

### 5.4. Interpretation of Ultrasound Features

Each ultrasound component of enthesitis has its own significance and relevance. The PD signal, a hallmark for active inflammation, showed substantial agreement (κ = 0.71) in the present study. The difference between images and direct assessment may be influenced by technical factors that affect the visibility of the signal, such as probe pressure or tendon tension, which may obscure weak signals. Moreover, the PD signal was assessed as a binary variable (present/absent). It is also possible that a minimal PD signal (e.g., grade 1), at the 2 mm limit from the cortical bone, may have been overlooked. Furthermore, in studies, some enthesitis ultrasound scores incorporating, for example, MASEI-PD or OMERACT-PD, demonstrated responsiveness to treatment in active PsA and SpA patients, improving after 3 and 6 months of therapy [29].

Structural changes such as enthesophytes and calcifications are often evaluated together in recent research and demonstrate strong inter-observer agreement due to their clear definition and visual distinction. Calcifications alone can be difficult to identify when close to the cortex. Erosions are highly specific for enthesitis in PsA Their detection also consistently shows high inter-observer agreement across all studies, as they can be reliably visualized on ultrasound in two planes.

Enthesial thickness remains difficult to evaluate in a standardized way. It must be measured at the junction between the tendon and enthesis and is usually compared to the contralateral side. As in previous studies, hypoechogenicity was the most challenging component to assess, showing poor inter-observer agreement. Another specific study on Achilles tendon enthesopathy showed, in a cohort of 28 patients with spondylarthritis, that hypoechogenicity was the most difficult element of enthesitis to detect [27].

### 5.5. The Achilles Tendon as a Target Site

For the present study, the Achilles tendon insertion on the calcaneus was chosen due to its superficial localization and easy ultrasound accessibility. The Achilles tendon was also the most relevant location for diagnosis, with erosions and PD activity at this level being the most discriminative features [15]. No PD signal or erosions were found in healthy controls. Also, DiMatteo et al. [28] demonstrated that among lower limb entheses, only the Achilles tendon was significantly associated with spondylarthritis, suggesting it should be the primary site for ultrasound evaluation, with further scanning places reserved for cases with normal findings. Interestingly, no association was found between OMERACT enthesitis features and DAPSA or the number of painful or swollen joints, suggesting these instruments measure different aspects of PsA with varied treatment responses.

In the OMERACT article on defining enthesitis [7], bursitis and tendinitis, with or without PD signal, were discussed but considered separate entities around the enthesis and were not included in the enthesitis definition. On the other hand, Benjamin, McGonagle et al. [30] first defined enthesitis histologically as a single organ, including the insertion, adipose tissue, fibrocartilage, bursa, adjacent trabecular bone, and deep fascia. McGonagle also authored the synovio-entheseal complex theory [31], which considers the bursa an integral part of the enthesis. According to this model, a patient with retrocalcaneal bursitis with PD signal and adjacent erosions could still reflect enthesis despite not fulfilling the current OMERACT criteria. Unlike OMERACT, the GUESS, one of the most used scores for ultrasound enthesitis [32], includes bursitis as a criterion for enthesitis.

### 5.6. False Positives and Confounding Conditions

DiMatteo et al. [20] detected ultrasound signs of enthesitis in a significant number of healthy subjects, emphasizing the risk of false-positive cases. In their study, various entheses were evaluated (e.g., patellar tendon insertions, Achilles tendon, and plantar fascia), showing that 34.1% of patients had inflammatory ultrasound findings per OMERACT in 8.4% of entheses, though only one enthesis (0.12%) showed a PD signal above grade 1 across 820 assessments.

In patients with metabolic syndrome, a high prevalence of ultrasound enthesitis was also observed (86%), with PD signal detected in 15% of these cases (1.52% of scanned entheses) [33]. Erosions were rare (0.3% of entheses). Also, male sex was associated with structural anomalies. PD signal correlated with LEI (r = 0.31), possibly reflecting vascular changes related to both inflammation and repair. Metabolic syndrome patients have a pro-inflammatory status, contributing to entheseal inflammation and pain.

Balint and Poddubny [34] suggested that structural changes (e.g., erosions) may help differentiate inflammatory from mechanical enthesitis. While the PD signal indicates active inflammation, its presence alone (without GS abnormalities) may not be sufficient to define inflammatory enthesitis. In both literature and the present study, erosions and PD signal (especially grade 2 or 3) were strongly associated with definite inflammatory enthesitis. These should be weighted accordingly in diagnostic decisions.

The findings of the present study are consistent with those reported by Ribeiro et al. [35], who evaluated Achilles tendon entheses using ultrasound in patients with psoriatic arthritis. In “possible” cases of enthesitis, PD grade 1 was observed at a much higher frequency (55.6%) compared to confirmed (“yes”) cases of inflammatory enthesitis (20.3%), suggesting that PD grade 1 contributes to diagnostic uncertainty, requiring additional features. In contrast, the presence of PD within erosions correlated strongly with confirmed (“yes”) inflammatory enthesitis, reinforcing the role of erosions as a structural marker.

### 5.7. Proposal for Future Research

This study proposes a combined score incorporating PD signal and erosions for diagnosing inflammatory enthesitis, which proved reliable and specific, though limited in sensitivity. The score may be validated either against a gold standard, such as magnetic resonance imaging (MRI), or through comparison with non-inflammatory control subjects. The challenge remains to develop and validate a PsA-specific ultrasound score for active inflammatory enthesitis that balances sensitivity and specificity. It is crucial to define when ultrasound inflammation is clinically relevant. Inflammatory enthesitis means more than just “active enthesitis”—it implies therapeutic action and treatment adjustment. Medical education and standardization are needed to improve diagnostic accuracy. Rapid training programs in entheseal ultrasound are recommended for residents and early-career clinicians to enhance diagnostic reliability and confidence [36].

### 5.8. Study Novelty and Limitations

The novelty of the present study lies in the evaluation of inter-observer agreement data on enthesitis components in PsA patients—a topic that remains unexplored. Agreement metrics (κ values) were calculated according to Light’s methodology for each enthesitis component, indicating the level of agreement among observers using standardized definitions, which is crucial for validating ultrasound as a diagnostic tool. In contrast, most PsA enthesitis studies focus on prevalence or clinical–ultrasound correlations rather than inter-observer agreement. Also, this study assesses MSUS inter-observer agreement specifically in a PsA population, while the prior studies generally focused on spondylarthritis. To increase the veracity of the results, we combined a direct inter-observer evaluation (real-time patient scanning by 4 expert sonographers) and also a retrospective image-based assessment (in 2 groups of 4 and 10 examiners). As a result, we obtained higher inter-observer agreement for several enthesitis components (erosions, enthesophytes, PD signal) than previously mentioned, reflecting training in OMERACT definition. The current study also aims to define consensus-driven diagnostic criteria for inflammatory enthesitis by consensus, using advanced ultrasound techniques, providing a valuable diagnostic and monitoring tool in PsA. This data supports the proposed ultrasound scoring system as a reliable tool for identifying inflammatory enthesitis in clinical and research settings.

The present study has several limitations that should be acknowledged. First, the small number of entheses evaluated reduces the statistical power of the findings. However, it is important to note that the study was conducted in a real-life setting, with simultaneous examination by four evaluators. As a pilot study, our intention was to explore inter-observer agreement using a tightly standardized MSUS protocol and to generate preliminary data that could guide further refinement of diagnostic criteria. Larger, prospectively designed studies across multiple centers are necessary in order to validate these findings and assess their applicability to broader PsA and SpA populations. Second, the evaluation was limited to a single entheseal site, the Achilles tendon, which may not adequately represent enthesitis at another anatomical site. Third, the lack of a control group, such as patients with Achilles tendon pain in the absence of an inflammatory rheumatic disease, limits the ability to differentiate between inflammatory and non-inflammatory diseases. Fourth, although patients were randomly selected across study days, recruitment was confined to a single center and over a short period, which may not fully capture the variability of PsA manifestations in broader clinical practice. Fifth, the “direct” and “recorded” inter-observer assessments, while complementary, introduce potential differences in examiner judgment when evaluating live scanning versus static images, particularly for dynamic features such as PD activity. Sixth, only rheumatologists with expertise in MSUS and specific training in the OMERACT definition participated in the study; agreement in less experienced examiners may differ. Finally, the statistical approach was limited to cross-sectional assessment and did not address longitudinal reproducibility or sensitivity to change, which are critical for validating ultrasound as an outcome measure in PsA enthesitis. Also, no formal a priori sample size calculation was performed before data collection. Post hoc estimations suggested that our recorded assessments were adequately powered, whereas the direct assessments were slightly short of the conservative target under worst-case category balance. This should be considered when interpreting the precision of the direct κ estimates.

## 6. Conclusions

Both direct and image-based inter-observer studies showed high agreement values for the following elements from the OMERACT definition of enthesitis: enthesophytes (κ > 0.6), erosions (κ > 0.5), and entheseal thickness (κ > 0.5). In both, low agreement was observed for hypoechogenicity (κ between 0.1 and 0.4), reinforcing its subjective and operator-dependent nature.

The relatively low inter-observer agreement regarding the presence of PD in the direct inter-observer study (κ = 0.35/0.45) highlights the need for targeted educational interventions aimed at improving the detection and interpretation of the PD signal in musculoskeletal ultrasound. Erosions and PD signal in erosions showed statistically significant differences between the “possible” and confirmed (“yes”) inflammatory enthesitis groups. A PD signal of grade 2 or 3 within the enthesis or erosions was observed exclusively in cases classified as definite (“yes”) inflammatory enthesitis. Similarly, a grade 3 PD signal in the bursa was found only in patients with confirmed (“yes”) inflammatory enthesitis.

This study proposes a novel ultrasound scoring system for defining inflammatory enthesitis, incorporating PD signal along erosions as a structural marker. The score demonstrated overall good diagnostic performance, with a sensitivity of 67% and a specificity of 100%.

## Figures and Tables

**Table 1:** Frequency and agreement for Achilles enthesitis in the direct inter-observer study for enthesitis definition (4 evaluators, 20 entheses assessed—GE machine).

Feature	Frequency	Light’s κ [95% CI]	κ Interpretation
Achilles enthesitis	58.8%	0.54 [0.51–0.87]	Moderate
Power Doppler	23.8%	0.45 [0.41–0.88]	Moderate
Hypoechogenicity	37.5%	0.10 [−0.37–0.56]	Poor
Thickening	52.5%	0.65 [0.63–0.92]	Substantial
Erosions	32.5%	0.73 [0.62–0.97]	Substantial
Enthesophytes	73.8%	0.63 [0.55–0.93]	Substantial
Calcifications	18.8%	0.34 [−0.23–0.92]	Fair
Bursitis	30.0%	0.27 [−0.22–0.76]	Fair
Normal	37.5%	0.72 [0.67–0.97]	Substantial

**Table 2:** Frequency and agreement for Achilles enthesitis in the recorded inter-observer study for enthesitis definition (4 evaluators, 20 entheses assessed—GE machine).

	Frequency	Light’s κ [95% CI]	κ Interpretation
Achilles enthesitis	51.3%	0.47 [0.46–0.84]	Moderate
Power Doppler	25.0%	0.71 [0.47–0.97]	Substantial
Hypoechogenicity	35.0%	0.14 [0.09–0.64]	Poor
Thickening	47.5%	0.52 [0.52–0.85]	Moderate
Erosions	33.8%	0.76 [0.44–0.97]	Substantial
Enthesophytes	50.0%	0.85 [0.66–0.97]	Substantial
Calcifications	10.0%	0.17 [0.03–0.49]	Poor
Bursitis	41.3%	0.60 [0.50–0.86]	Moderate
Normal	18.8%	0.48 [0.42–0.77]	Moderate

**Table 3:** Proposed scoring system for classifying inflammatory enthesitis.

Ultrasound Element	Points
PD signal in enthesis > 1	2
PD signal in enthesis = 1	1
At least one erosion	1
PD signal in erosion > 1	1
PD signal in bursa > 2	1

Note: A total score of at least 2 defines inflammatory enthesitis.

## Data Availability

Dataset available on request from the authors.

## Abbreviations

The following abbreviations are used in this manuscript:

bDMARDs	Biologic Disease-Modifying Antirheumatic Drugs
csDMARDs	Conventional Synthetic Disease-Modifying Antirheumatic Drugs
EFSUMB	European Federation of Societies for Ultrasound in Medicine and Biology
EULAR	European Alliance of Associations for Rheumatology
GUESS	Glasgow Ultrasound Enthesitis Scoring System
MASEI-PD	Madrid Sonographic Enthesitis Index—Power Doppler
OMERACT	Outcome Measures in Rheumatology
OMERACT-PD	Outcome Measures in Rheumatology—Power Doppler
